# Outcomes in Patients with Acute and Stable Coronary Syndromes; Insights from the Prospective NOBORI-2 Study

**DOI:** 10.1371/journal.pone.0088577

**Published:** 2014-02-14

**Authors:** Farzin Fath-Ordoubadi, Erik Spaepen, Magdi El-Omar, Douglas G. Fraser, Muhammad A. Khan, Ludwig Neyses, Gian B. Danzi, Ariel Roguin, Dragica Paunovic, Mamas A. Mamas

**Affiliations:** 1 Manchester Heart Centre, Manchester Royal Infirmary, Manchester, United Kingdom; 2 SBD Analytics, Hertstraat, Bekkevoort, Belgium; 3 Division of Cardiology, Fondazione IRCCS Cà Granda, Ospedale Maggiore Policlinico, Milan, Italy; 4 Department of Cardiology, Rambam Medical Center, Haifa, Israel; 5 European Medical and Clinical Division, Terumo Europe, Leuven, Belgium; 6 Cardiovascular Institute, University of Manchester, Manchester, United Kingdom; College of Pharmacy, University of Florida, United States of America

## Abstract

**Background:**

Contemporary data remains limited regarding mortality and major adverse cardiac events (MACE) outcomes in patients undergoing PCI for different manifestations of coronary artery disease.

**Objectives:**

We evaluated mortality and MACE outcomes in patients treated with PCI for STEMI (ST-elevation myocardial infarction), NSTEMI (non ST-elevation myocardial infarction) and stable angina through analysis of data derived from the Nobori-2 study.

**Methods:**

Clinical endpoints were cardiac mortality and MACE (a composite of cardiac death, myocardial infarction and target vessel revascularization).

**Results:**

1909 patients who underwent PCI were studied; 1332 with stable angina, 248 with STEMI and 329 with NSTEMI. Age-adjusted Charlson co-morbidity index was greatest in the NSTEMI cohort (3.78±1.91) and lowest in the stable angina cohort (3.00±1.69); P<0.0001. Following Cox multivariate analysis cardiac mortality was independently worse in the NSTEMI vs the stable angina cohort (HR 2.31 (1.10–4.87), p = 0.028) but not significantly different for STEMI vs stable angina cohort (HR 0.72 (0.16–3.19), p = 0.67). Similar observations were recorded for MACE (<180 days) (NSTEMI vs stable angina: HR 2.34 (1.21–4.55), p = 0.012; STEMI vs stable angina: HR 2.19 (0.97–4.98), p = 0.061.

**Conclusions:**

The longer-term Cardiac mortality and MACE were significantly worse for patients following PCI for NSTEMI even after adjustment of clinical demographics and Charlson co-morbidity index whilst the longer-term prognosis of patients following PCI STEMI was favorable, with similar outcomes as those patients with stable angina following PCI.

## Introduction

Percutaneous coronary intervention (PCI) has become the revascularisation therapy of choice in patients with both stable coronary artery disease and acute coronary syndromes. During the past few decades, multiple randomised controlled trials have been undertaken to assess the efficacy of both pharmacological, stent technology and adjunctive device developments on morbidity and mortality in both stable and acute coronary syndrome subgroups of patients [1], [2]. However, despite this, contemporary data remains limited regarding mortality and major adverse cardiac events (MACE) outcomes when comparing across the spectrum of patients with different indications for PCI in a “real-life” setting. For example, similar in-hospital mortality rates have been described in non ST-elevation myocardial infarction (NSTEMI) and ST-elevation myocardial infarction (STEMI) in some studies [3], [4] whilst others have reported higher mortality rates amongst patients with STEMI [5], [6]. In the longer term, some studies have suggested that the prognosis was worse in STEMI as compared to NSTEMI [7]. Other studies have reported the opposite in the long term [6] and only few studies have compared the outcome of these patient groups to those undergoing elective PCI [6]. Studies that have compared outcomes between STEMI and NSTEMI cohorts are often difficult to interpret since a significant proportion of NSTEMI patients may not have received revascularisation in these studies whilst the majority of patients presenting with STEMI do [4], [7]. Furthermore, in those studies bare-metal stent (BMS) and drug eluting stent (DES) usage which is known to influence MACE rates varies significantly amongst stable and acute coronary syndrome subgroups of patients [3], [8]. This could further impact outcomes when comparing across the spectrum of patients with different indications for PCI in a “real-life” setting.

We have therefore evaluated early and late mortality and MACE outcomes in patients who have been treated with PCI for STEMI, NSTEMI and stable angina in an all-comer population through analysis of data derived from a large prospective multicenter study conducted in 125 centres across Europe and Asia using only DES - the Nobori-2 study.

## Methods

### Study Design and Patient Population

Nobori 2 is a prospective, multicenter study conducted in 125 centres across Europe and Asia to investigate the performance of the Nobori DES system in an all-comers clinical setting [9] with the only exclusion criterion used being the patient’s refusal or inability to provide written informed consent. All patients that had at least one Nobori DES implanted or attempted were included in the analysis. All patients signed informed consent form reviewed and approved by the Institutional Review Board or Ethics Committee of each participating centres. Outcomes were stratified by indication for PCI; Stable Angina, NSTEMI and STEMI. Patients presenting with unstable angina were pooled with the NSTEMI cohort.

### Outcomes and Study Definition

ACS was defined as typical symptoms with ischemic electrocardiographic changes including ST-segment elevation and non–ST-segment elevation and/or laboratory evidence of myocardial damage. All clinical, demographic and outcome data were collected into a Web-based data management system coordinated and analyzed by independent companies (KIKA Medical, Paris, France, and SBD Analytics, Bekkevoort, Belgium, respectively). Clinical follow-up data included the documentation of adverse events, in death, MI, repeat revascularisation, stent thrombosis, bleeding and angina status.

Follow-up was performed at 1 month, 6 months, and 12 months, and yearly up to 5 years. All clinical end points were adjudicated by an independent clinical events committee. Twelve-month follow-up rate was 97% and at 2-years was 95%. The primary end point was cardiac mortality. MACE were defined as a composite of cardiac death, myocardial infarction (MI) and target vessel revascularization (TVR).

### Statistical Analysis

Continuous variables are presented as mean±standard deviation and were compared using the non-parametric tests: the Kruskal-Wallis test to compare multiple groups (>2). All tests were 2-sided. Categorical variables are presented as frequencies and percentages, and were compared using Cochran–Mantel-Haenszel test or Fisher’s exact test. Kaplan–Meier estimates were generated, and comparisons of MACE and mortality events were made using log-rank test. Cox proportional hazards regression was used to assess pair-wise hazard ratios (HR) of the 3 subgroups under investigation, either unadjusted (no other covariates) or adjusted for some selected covariates. The censoring time of a patient for these time-to-event analyses was defined as the patient’s last observation time, i.e. follow-up or event time. The proportionality assumption for the Cox regression models was tested using the Supremum Test and cumulative score process plots (Cumulative martingale residuals). In case the proportional hazards assumption was violated for the main covariate (ACS status), the covariate was appropriately made time-dependent to maintain proportionality. Data analysis was performed by an independent statistical office (SBD Analytics, Bekkevoort, Belgium), using the statistical software package SAS V8.2 (The SAS Institute, Cary, NC).

## Results

The Nobori-2 trial enrolled patients from 125 centres across the world and 1909 patients were included in this analysis. A total of 1332 patients who underwent PCI had a diagnosis of stable angina (69.7%) whilst 577 patients were diagnosed with ACS (30.3%). 248 of the patients with ACS presented with STEMI (43%) whilst 329 patients presented with NSTEMI (57%). Clinical demographics are presented in Table 1. The patients presenting with STEMI were significantly younger than those presenting with NSTEMI or stable angina and the age adjusted Charlson co-morbidity index was greatest in the NSTEMI cohort and lowest in the stable angina cohort.

**Table 1:** Clinical Demographics.

Variable	Angina(n = 1,332)	NSTEMI (n = 329)	STEMI (n = 248)	P-Value
Age (mean ±SD)	64.4±10.5	65.0±11.8	61.3±11.8	<0.0001
Gender (% Male)	1023 (76.8%)	252 (76.6%)	194 (78.2%)	0.89
Hypercholesterolaemia	993 (74.5%)	220 (66.9%)	126 (50.8%)	<0.0001
Hypertension	996 (74.8%)	219 (66.6%)	119 (48.0%)	<0.0001
Diabetes	379 (28.5%)	99 (30.1%)	66 (26.6%)	0.64
Smoker	220 (16.5%)	101 (30.7%)	109 (44.0%)	<0.0001
History of Heart Failure	41 (3.1%)	13 (4.0%)	5 (2.0%)	0.47
Previous AMI	429 (32.2%)	113 (34.3%)	91 (36.7%)	0.35
Previous PCI	487 (37.2%)	70 (21.3%)	29 (11.7%)	P<0.0001
Charlson score (mean ±SD)	3.00±1.69	3.78±1.91	3.21±1.66	P<0.0001
*Charlson score (mean ±SD)	1.06±1.19	1.78±1.31	1.54±0.95	P<0.0001

*(without age scoring).

Procedural demographics are presented in Table 2, which demonstrates that the mean number of lesions treated, mean stent length and mean number of stents was similar across all 3 groups. Table 3 illustrates lesion characteristics and QCA analysis of lesions pre- and post-treatment. Lesion characteristics and type were similar across the 3 cohorts studied.

**Table 2:** Procedural Demographics.

Variable	Angina (n = 1,332)	NSTEMI (n = 329)	STEMI (n = 248)	P-Value
Glycoprotein IIb/IIIa	185 (14.7%)	92 (27.9%)	98 (39.5%)	0.0001
Radial Access	439 (33.2%)	145 (44.2%)	89 (35.8%)	0.001
Number of vessels diseased	1.73±0.78	1.77±0.75	1.68±0.72	0.42
Number of vessels treated	1.23±0.48	1.26±0.48	1.28±0.53	0.27
Number of lesions detected	1.97±1.11	2.10±1.11	2.01±1.07	0.076
Number of lesions treated	1.44±0.77	1.46±0.71	1.48±0.80	0.62
Number of stents	1.73±1.10	1.71±0.98	1.82±1.19	0.68
Stent Length	33.44±22.28	32.48±19.94	33.09±38.95	0.15

**Table 3:** Lesion data (data presented per lesion.

Variable	Angina (n = 1,916)	NSTEMI (n = 479)	STEMI (n = 368)	P-Value
**Target Vessel**				
RCA	596 (31.1%)	128 (26.7%)	131 (35.6%)	0.021
LAD	746 (38.9%)	186 (38.8%)	167 (45.4%)	0.063
LCx	515 (26.9%)	150 (31.3%)	64 (17.4%)	<0.0001
Left Main	31 (1.62%)	3 (0.63%)	3 (0.82%)	0.199
SVG	28 (1.46%)	12 (2.51%)	3 (0.82%)	0.132
**Lesion Characteristics**				
	**(n = 1,661)**	**(n = 438)**	**(n = 337)**	
Ostial lesion	181 (10.9%)	49 (11.2%)	22 (6.5%)	0.037
Bifurcation	329 (19.8%)	87 (19.9%)	52 (15.4%)	0.163
Tortuous	131 (7.9%)	38 (8.7%)	17 (5.05%)	0.122
Calcified	432 (26.0%)	102 (23.3%)	85 (25.2%)	0.518
**Lesion Type**				
A	63 (3.8%)	13 (3.0%)	8 (2.4%)	0.404
B1	403 (24.3%)	93 (21.3%)	79 (23.4%)	0.44
B2	687 (41.3%)	193 (44.2%)	107 (31.8%)	0.001
C	508 (30.6%)	138 (31.6%)	142 (42.1%)	0.0002
**QCA Results Pre**				
Ref vessel diam (mm)	2.61±0.60 (1,528)	2.64±0.55 (398)	2.61±0.58 (252)	0.436
MLD (mm)	0.87±0.50 (1,655)	0.76±0.45 (436)	0.61±0.52 (335)	<0.0001
Lesion Length (mm)	15.61±9.93 (1,528)	16.19±8.66 (398)	16.44±9.71 (252)	0.0504
Diameter stenosis (%)	66.81±17.24 (1,655)	71.27±16.29 (437)	76.52±18.95 (335)	<0.0001
**QCA Results Post**				
Ref vessel diam (mm)	2.89±0.51 (1,604)	2.87±0.50 (429)	2.93±0.49 (321)	0.238
MLD (mm)	2.51±0.47 (1,604)	2.50±0.47 (429)	2.54±0.47 (321)	0.686
Stenosis in stent (%)	13.07±6.77 (1,604)	13.03±7.44 (429)	13.42±7.23 (321)	0.668

**Figure 1:**
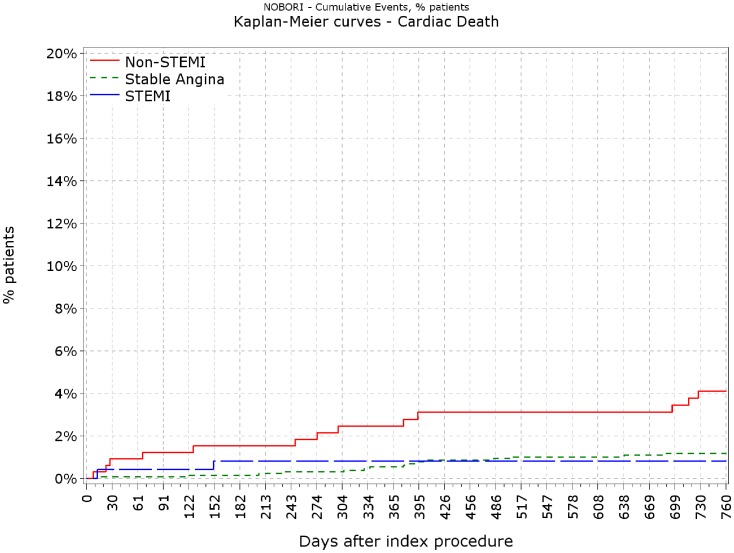
Kaplan-Meier curve for cardiac death.

**Figure 2:**
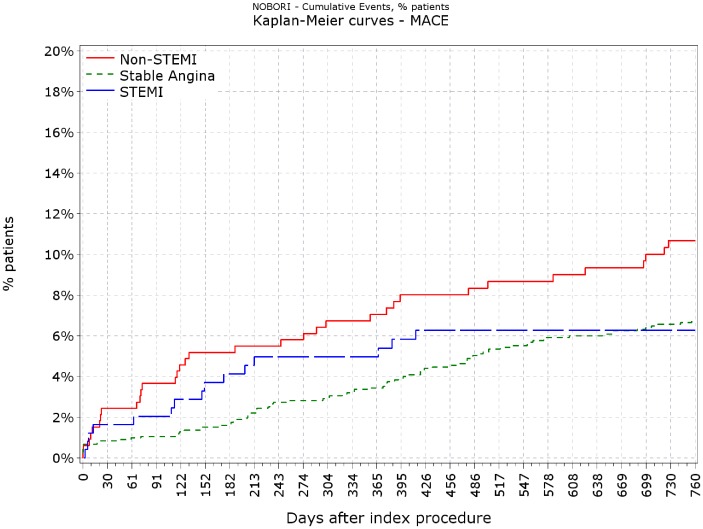
Kaplan- Meier curves for MACE.

Figure 1 illustrates Kaplan-Meier unadjusted survival curves for cardiac death for all 3 cohorts. A statistically significant increase in cardiac death was observed in the NSTEMI cohort compared to the stable angina cohort (unadjusted HR 3.17, 95% CI 1.54–6.53, p = 0.0017) whereas survival was not statistically different the STEMI group compared to the stable angina group (unadjusted HR 0.64 95%CI 0.15–2.78, p = 0.55). Figure 2 illustrates Kaplan-Meier unadjusted survival curves for MACE for all 3 cohorts. As the proportionality assumption was violated for the Cox model with MACE as outcome, Process Score plots were created. These indicated that a time cut-off around 180 days would reintroduce proportionality. That is, assessing the effects of ACS status before and after 180 days separately (but simultaneously model), will yield valid estimates for each of the time categories, for the ACS status. Similarly, a statistically significant increase in MACE was observed in the NSTEMI cohort compared to the stable angina cohort (unadjusted HR (≤180 days) 3.16, 95% CI 1.70–5.96; P = 0.0004) whereas MACE was not significantly different in the STEMI group compared to the stable angina group (unadjusted HR (≤180 days) 5.44 95% CI 0.77–38.67; P = 0.09).

Table 4 illustrates mortality and MACE events for the stable angina, NSTEMI and STEMI groups at 30 days, 6 months, 1 year and 2 years. It can be seen that unadjusted 30-day cardiac mortality rates were higher in the NSTEMI and STEMI groups compared to the stable angina cohort (0.91%, 0.40% and 0.08% respectively; P = 0.021), although by two years cardiac mortality was similar in the STEMI and stable angina cohort but remained increased in the NSTEMI group (1.13%, 0.81% and 3.95% respectively; P = 0.0021). Similarly, 30-day unadjusted MACE events were greater in the NSTEMI and STEMI cohorts at baseline (2.4%, 1.6% compared to 0.8% in stable angina cohort; P = 0.039) although by 2 years follow up MACE events were similar in the stable angina and STEMI cohort but remained worse in the NSTEMI group (6.5%, 6.8% and 10.3% respectively; P = 0.048).

**Table 4:** Clinical outcomes.

Timepoint	Angina(n = 1,332)	NSTEMI(n = 329)	STEMI(n = 248)	P-Value
**Cardiac Mortality**				
30-Day	1 (0.08%)	3 (0.91%)	1 (0.4%)	0.021
6 month	2 (0.15%)	5 (1.52%)	2 (0.81%)	0.0041
1 year	10 (0.75%)	10 (3.04%)	2 (0.81%)	0.0044
2 years	15 (1.13%)	13 (3.95%)	2 (0.81%)	0.0021
**MACE**				
30-Day	11 (0.8%)	8 (2.4%)	4 (1.6%)	0.0393
6 month	25 (1.9%)	17 (5.2%)	10 (4.0%)	0.0022
1 year	51 (3.8%)	26 (7.9%)	14 (5.7%)	0.008
2 years	86 (6.5%)	34 (10.3%)	15 (6.1%)	0.048
**Myocardial Infarction**				
30-Day	10 (0.8%)	6 (1.8%)	3 (1.2%)	0.164
6 month	11 (0.8%)	10 (3.0%)	5 (2.0%)	0.0039
1 year	17 (1.3%)	15 (4.6%)	5 (2.0%)	0.0012
2 years	27 (2.0%)	17 (5.2%)	6 (2.4%)	0.01
**Target vessel revascularisation**				
30-Day	2 (0.2%)	3 (0.9%)	2 (0.8%)	0.0328
6 month	14 (1.1%)	8 (2.4%)	7 (2.8%)	0.0288
1 year	32 (2.4%)	13 (4.0%)	11 (4.4%)	0.10
2 years	56 (4.2%)	18 (5.5%)	11 (4.4%)	0.57

Multivariate analysis, using Cox regression, adjusted for clinical demographics and Charlson score for co-morbidity was performed for cardiac mortality and MACE events and this is summarized in Table 5. This demonstrates that after multivariate adjustment, NSTEMI was independently associated with worse cardiac mortality compared to the stable angina cohort following adjustment of baseline clinical demographics and Charlson co-morbidity score, whilst cardiac mortality and MACE were not significantly different in the STEMI cohort when compared to the stable angina cohort.

**Table 5:** Unadjusted and adjusted Hazard Ratios and for cardiac death and MACE.

Endpoint	Unadjusted OR (95% CI)	Age, Gender adjustedOR (95% CI)	* Fully adjusted OR (95% CI)
**Cardiac Mortality**			
NSTEMI vs Stable Angina	3.17 (1.54–6.53), p = 0.0017**	2.84 (1.38–5.87), p = 0.0049**	2.31 (1.10–4.87), p = 0.028**
STEMI vs Stable Angina	0.64 (0.15–2.78), p = 0.55	0.75 (0.17–3.26), p = 0.70	0.72 (0.16–3.19), p = 0.67
NSTEMI vs STEMI	4.92 (1.11–21.74), p = 0.035**	3.77 (0.85–16.66), p = 0.081	3.21 (0.71–14.50), p = 0.13
**MACE** ***			
**≤180 days**			
NSTEMI vs Stable Angina	3.16 (1.68–5.96), p = 0.0004**	3.06 (1.63–5.76), p = 0.0005**	2.34 (1.21–4.55), p = 0.012**
STEMI vs Stable Angina	2.49 (1.18–5.26), p = 0.017**	2.75 (1.30–5.82), p = 0.008*	2.19 (0.97–4.98), p = 0.061
NSTEMI vs STEMI	1.27 (0.58–2.78), p = 0.55	1.11 (0.51–2.43), p = 0.79	1.07 (0.45–2.54), p = 0.88
**>180 days**			
NSTEMI vs Stable Angina	1.07 (0.63–1.83), p = 0.80	1.04 (0.61–1.78), p = 0.87	0.86 (0.50–1.50), p = 0.60
STEMI vs Stable Angina	0.415 (0.17–1.03), p = 0.058	0.45 (0.18–1.13), p = 0.088	0.46 (0.18–1.14), p = 0.094
NSTEMI vs STEMI	2.59 (0.96–7.01), p = 0.062	2.30 (0.85–6.23), p = 0.10	1.89 (0.69–5.18), p = 0.22

OR corresponds to odds ratio,

*Adjusted for age, gender, hypertension, hypercholesterolaemia, diabetes and Charlson Index.

**equates to statistical significance.

***Time-dependent parameterization of ACS classification for MACE due to non-proportionality - cutoff at 180d.

## Discussion

The current analysis was undertaken in patients undergoing PCI for different manifestations of coronary artery disease such as high risk acute coronary syndromes (STEMI and NSTEMI) and stable angina in an all-comer population through analysis of data derived from a prospective multicenter study conducted in 125 centres across Europe and Asia using a single DES platform. The main findings of the study were that cardiac mortality and MACE outcomes of patients following PCI for NSTEMI were significantly worse than patients undergoing PCI for stable angina, even after adjustment for baseline clinical demographics and comorbidities using the Charlson co-morbidity score, whereas longer cardiac mortality and MACE outcomes of patients following PCI for STEMI were similar to those following PCI with stable angina following adjustment for baseline clinical demographics and co-morbidities.

To our knowledge this is one of the first studies that has compared short and longer-term outcomes in patients undergoing PCI for different manifestations of coronary artery disease using a single drug eluting stent platform. Previous studies have shown that in-hospital mortality rates have been greater in patients presenting with STEMI than those with NSTEMI [6], [7], [10], [11] whilst other studies have reported similar in-hospital mortality rates [4], [12]. Similarly at 6 years follow up mortality was greater in patients presenting with NSTEMI compared to those patients presenting with STEMI or stable angina in the study of Hirsch et al [6]. Other studies have shown either worse outcomes in NSTEMI cohort [11]–[13] or similar outcomes in STEMI and NSTEMI patients on longer term follow up [4]. Interpretation of many of these previous studies is complicated by the observation that they included patients with NSTEMI and STEMI acute coronary syndromes who were managed by both PCI or conservative treatment strategies [4], [7], [12] with significant differences in PCI rates in each respective cohort [4], [7], [12]. Such differences in the respective revascularisation rate amongst NSTEMI and STEMI patients has been shown to have significant implications on longer terms outcomes [7] and so would significantly bias outcomes previously reported for NSTEMI vs STEMI cohorts. Furthermore, interpretation of previous studies comparing outcomes between NSTEMI, STEMI and stable angina cohorts following PCI are complicated by the fact that there were significant differences in DES/BMS use between the cohorts studied which will impact on outcomes [6]. For example, DES use was infrequent in the study of Hirsch et al. [6] (STEMI cohort 1%, NSTEMI 8% and stable angina 11%) with the majority of PCI procedures undertaken with BMS platforms which is not reflective of contemporary PCI practice where use of drug eluting stent platforms are much more widespread.

Our findings of worse cardiac mortality and MACE outcomes associated with patients undergoing PCI for NSTEMI compared to those with stable angina, with similar longer term MACE and mortality outcomes in the STEMI vs stable angina cohorts undergoing PCI is of interest. Whilst patients with NSTEMI undergoing PCI were older compared to both the STEMI and stable angina cohorts, which would in itself lend to worse outcomes in the NSTEMI cohort, the association between NSTEMI and adverse outcomes persisted even after multi-variate adjustment for age. Patients presenting with NSTEMI often have a higher prevalence of cardiovascular and non-cardiovascular co-morbidities compared to patients with STEMI [4], [5], [12], [14], [15] and the presence of such unmeasured confounders has been suggested to contribute to the adverse outcomes associated with NSTEMI in previous studies. We have also confirmed that patients presenting with NSTEMI have a greater prevalence of co-morbid conditions compared to the STEMI and stable angina cohorts as evidenced by the greater Charlson co-morbidity score in the NSTEMI cohort. The Charlson co-morbidity score has been shown to be an important independent predictor of mortality [16], stent thrombosis and major bleeding [17] in patients undergoing PCI. However, even following adjustment for the presence of co-morbidities through inclusion of the Charlson score in our multivariate analysis, NSTEMI was independently associated with worse cardiac mortality. The worse cardiac mortality outcomes associated with NSTEMI may relate to residual confounders that we may not have measured in the older NSTEMI group such as more severe coronary artery disease in non-revascularised areas of the coronary vasculature, greater frailty that is a strong predictor of mortality outcomes following PCI [16] or a greater prevalence of unmeasured co-morbid conditions that are not included in the Charlson co-morbidity score.

Whilst the current analysis provides insights into outcomes of patients undergoing PCI for different manifestations of coronary artery disease such as ACS (STEMI and NSTEMI) and stable angina, the findings of our study are not applicable to patients with stable angina or an ACS who are managed with a non-invasive strategy. Often these patients are more elderly and have significantly more cardiovascular and non-cardiovascular co-morbidities and so may have worse outcomes than reported here [7]. Indeed, an invasive PCI strategy was independently associated with a 36% and 49% reduction in 2- year mortality in NSTEMI and STEMI groups in the study of Polonski et al [7]. Secondly, information regarding the medical treatment of patients in the current analysis was not available and so we are unable to comment on adherence to evidence based therapies in these cohorts and so are unable to assess the influence of medical therapy on long-term outcomes. Thirdly, Due to the observational character of this study and the multitude of analyses performed, it was not feasible to adjust for multiple testing. As such, we have supplied nominal p-values, not adjusted for multiple testing. Finally, the possibility of selection bias cannot be excluded, as the patients were not consecutively recruited at the study centres.

In conclusion, current analysis undertaken in patients undergoing PCI for different manifestations of coronary artery disease such as acute coronary syndromes (STEMI and NSTEMI) and stable angina in an all-comer (“real world”) population has shown that NSTEMI presentation is associated with adverse cardiac mortality and MACE.
